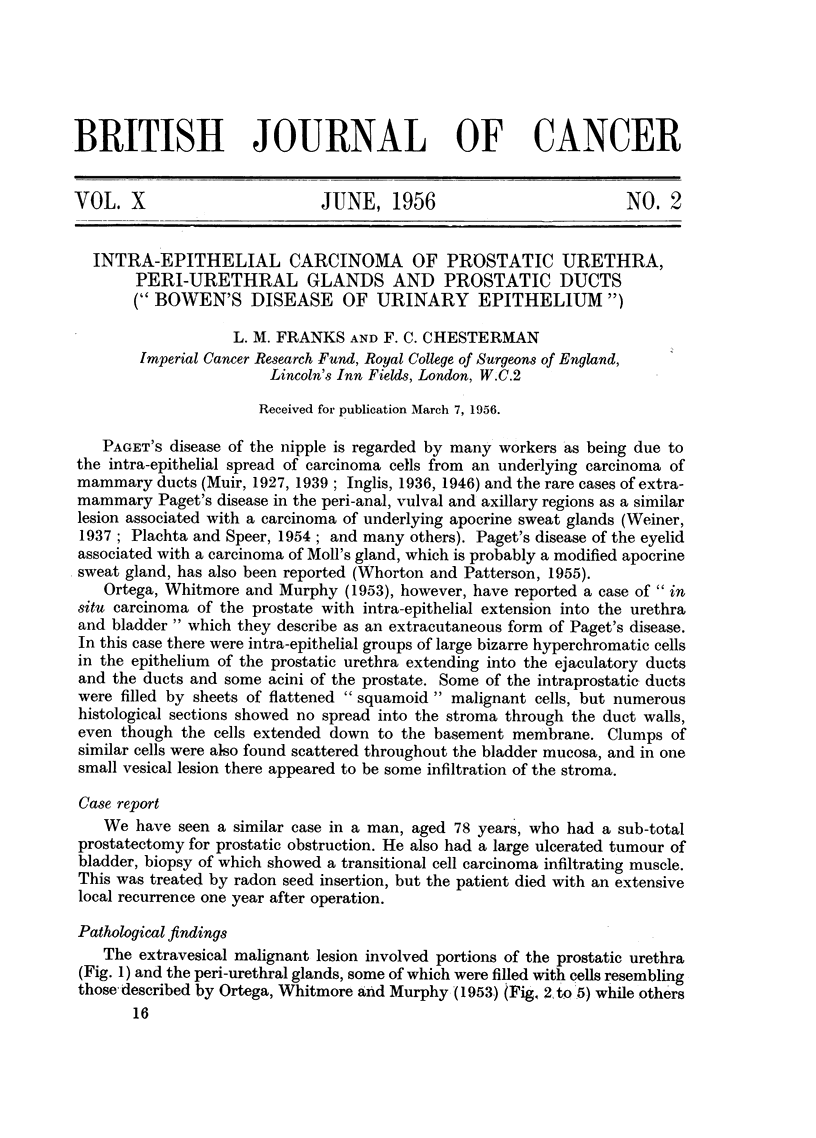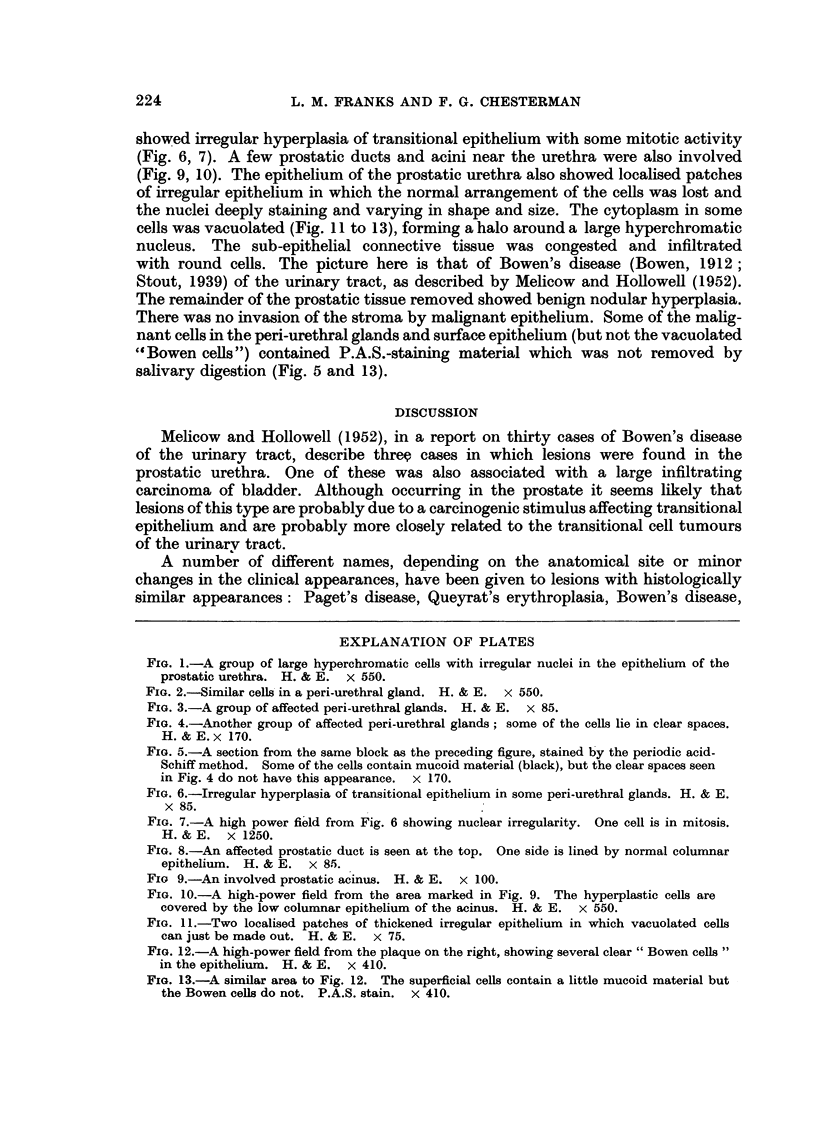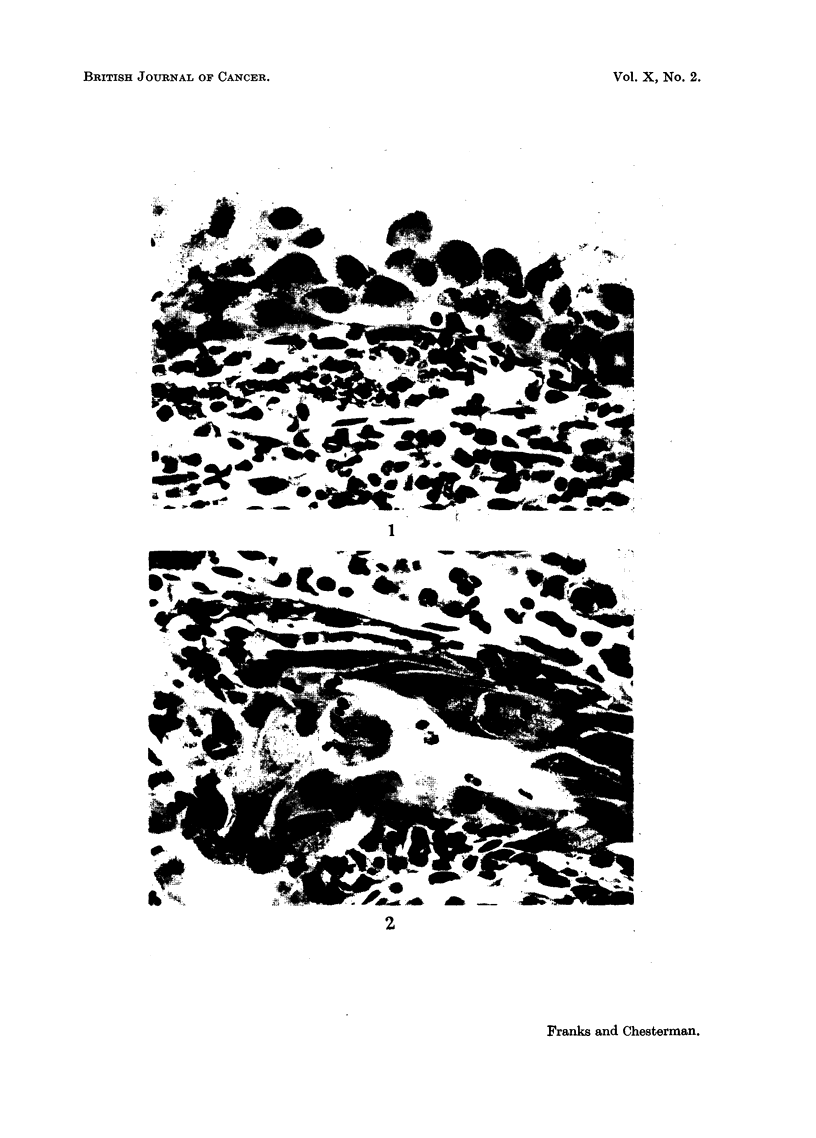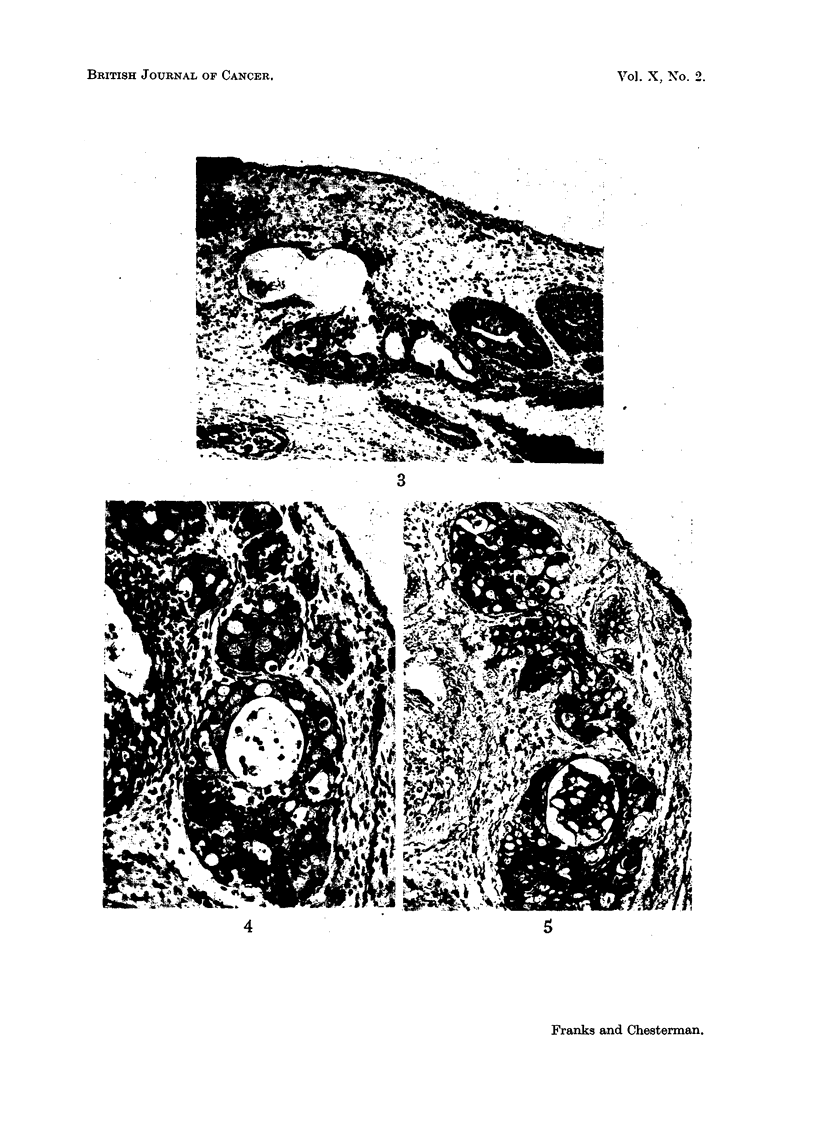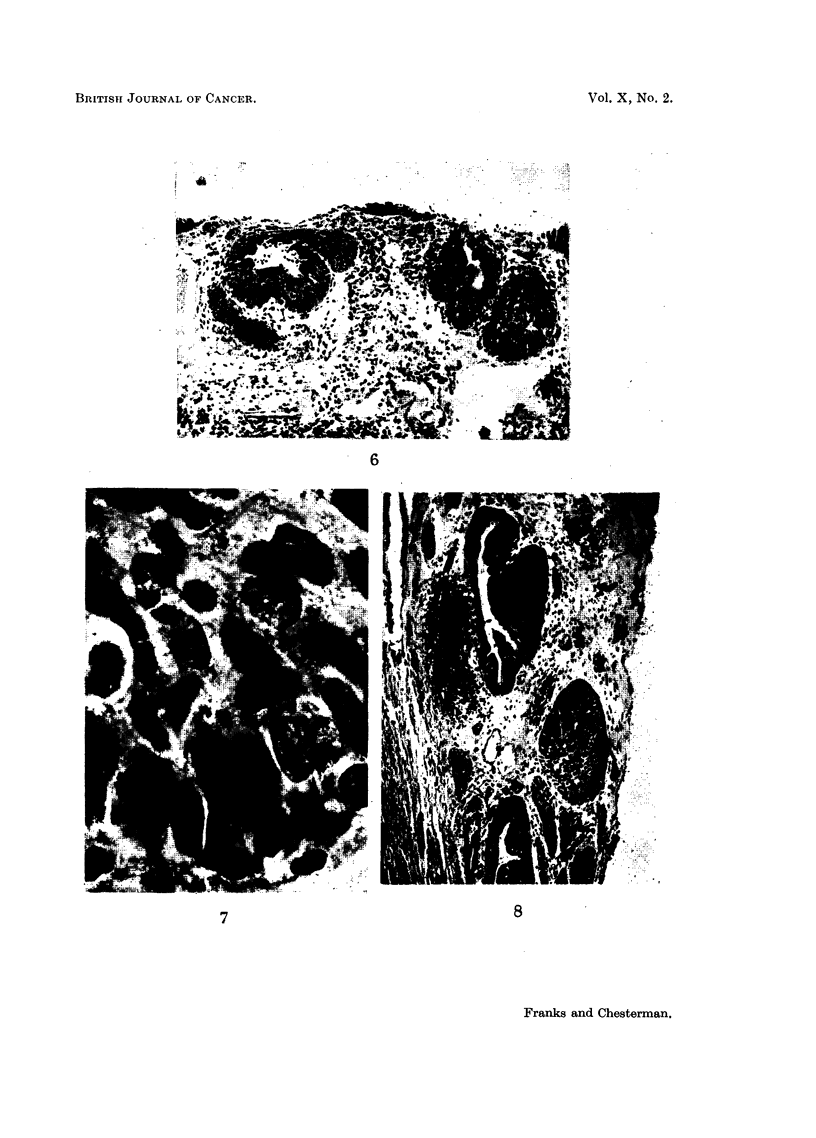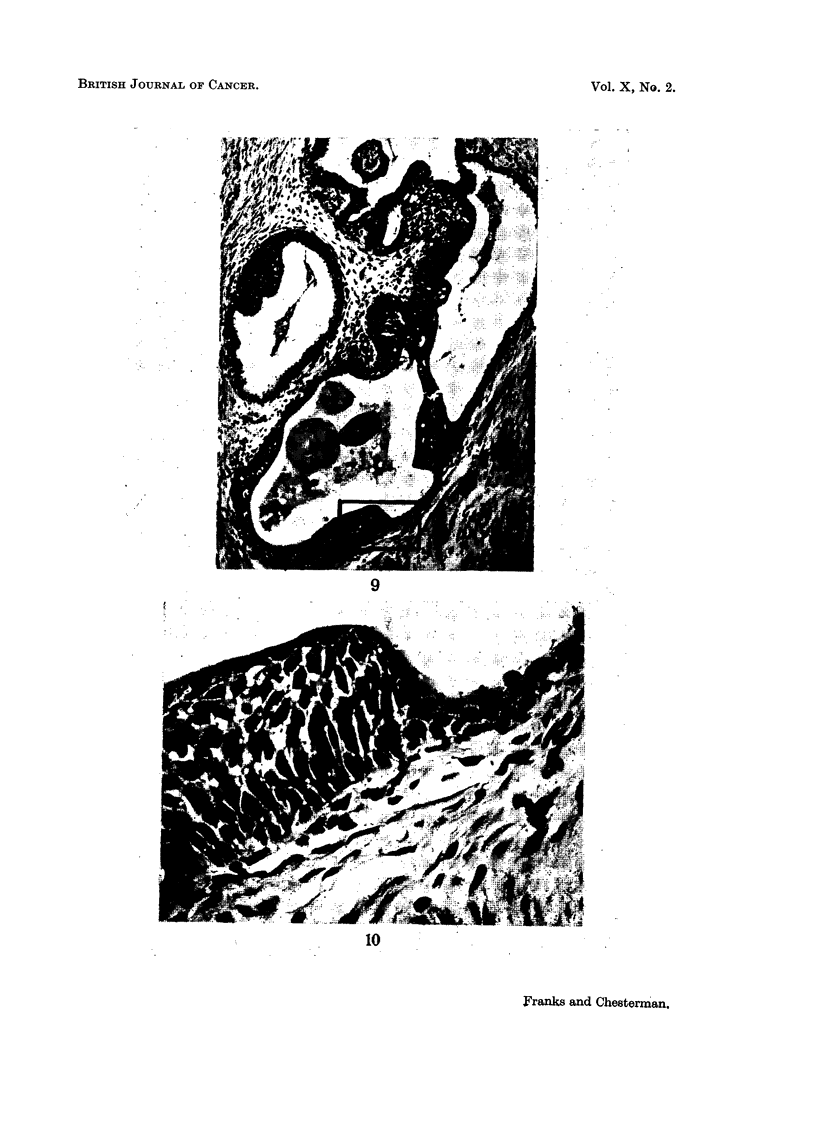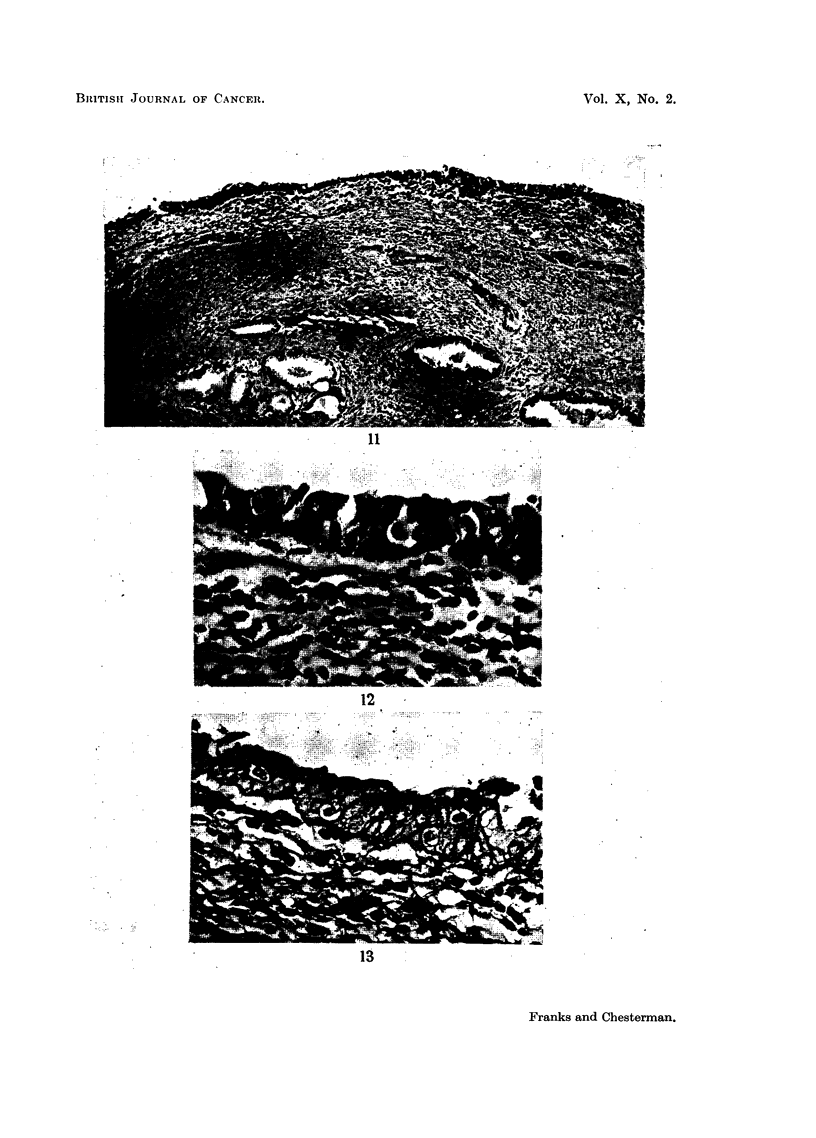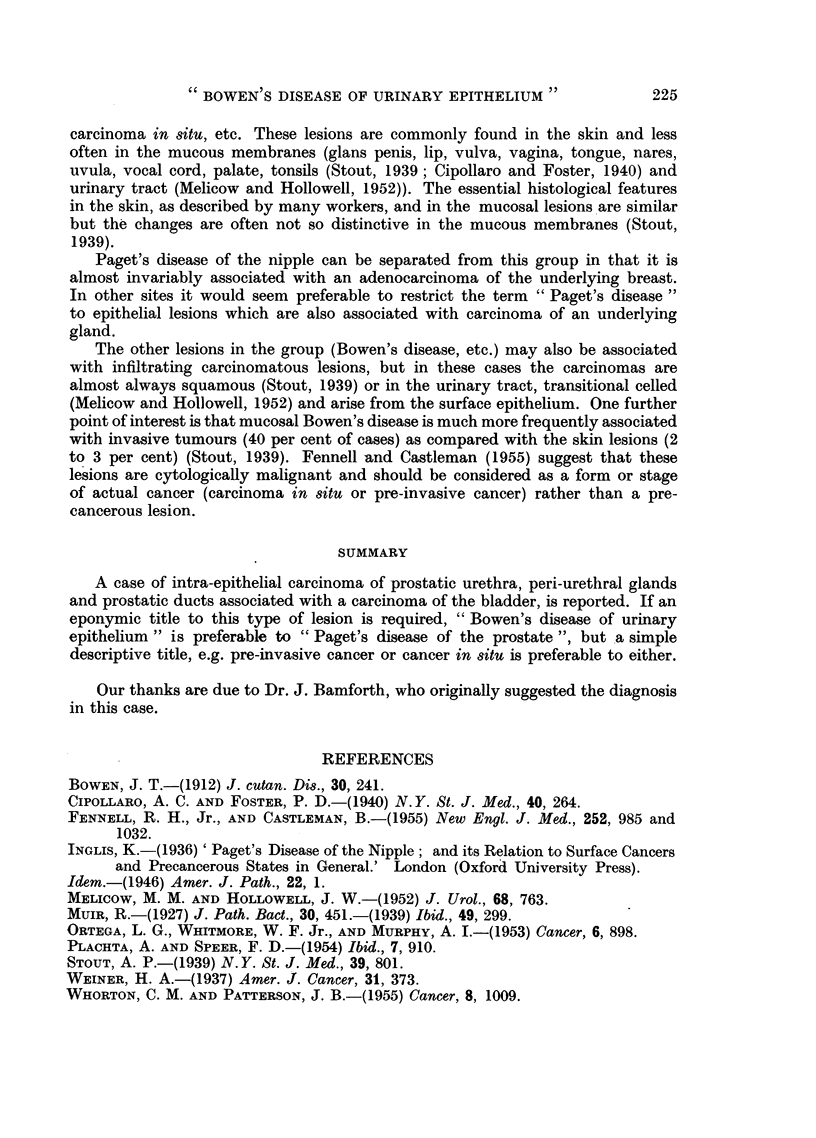# Intra-Epithelial Carcinoma of Prostatic Urethra, Peri-Urethral Glands and Prostatic Ducts (“ Bowen's Disease of Urinary Epithelium ”)

**DOI:** 10.1038/bjc.1956.25

**Published:** 1956-06

**Authors:** L. M. Franks, F. C. Chesterman

## Abstract

**Images:**


BRITISH JOURNAL OF CANCER

VOL. X JUNE, 1956 NO. 2

### INTRA-EPITHELIAL CARCINOMA OF PROSTATIC URETHRA, PERI-URETHRAL GLANDS AND PROSTATIC DUCTS ("BOWEN'S DISEASE OF URINARY EPITHELIUM")

L. M. FRANKS AND F. C. CHESTERMAN

*Imperial Cancer Research Fund, Royal College of Surgeons of England, Lincoln's Inn Fields, London, W.C.2*

Received for publication March 7, 1956.

PAGET'S disease of the nipple is regarded by many workers as being due to the intra-epithelial spread of carcinoma cells from an underlying carcinoma of mammary ducts (Muir, 1927, 1939 ; Inglis, 1936, 1946) and the rare cases of extra-mammary Paget's disease in the peri-anal, vulval and axillary regions as a similar lesion associated with a carcinoma of underlying apocrine sweat glands (Weiner, 1937 ; Plachta and Speer, 1954 ; and many others). Paget's disease of the eyelid associated with a carcinoma of Moll's gland, which is probably a modified apocrine sweat gland, has also been reported (Whorton and Patterson, 1955).

Ortega, Whitmore and Murphy (1953), however, have reported a case of "*in situ* carcinoma of the prostate with intra-epithelial extension into the urethra and bladder" which they describe as an extracutaneous form of Paget's disease. In this case there were intra-epithelial groups of large bizarre hyperchromatic cells in the epithelium of the prostatic urethra extending into the ejaculatory ducts and the ducts and some acini of the prostate. Some of the intraprostatic ducts were filled by sheets of flattened "squamoid" malignant cells, but numerous histological sections showed no spread into the stroma through the duct walls, even though the cells extended down to the basement membrane. Clumps of similar cells were also found scattered throughout the bladder mucosa, and in one small vesical lesion there appeared to be some infiltration of the stroma.

*Case report*

We have seen a similar case in a man, aged 78 years, who had a sub-total prostatectomy for prostatic obstruction. He also had a large ulcerated tumour of bladder, biopsy of which showed a transitional cell carcinoma infiltrating muscle. This was treated by radon seed insertion, but the patient died with an extensive local recurrence one year after operation.

*Pathological findings*

The extravesical malignant lesion involved portions of the prostatic urethra (Fig. 1) and the peri-urethral glands, some of which were filled with cells resembling those described by Ortega, Whitmore and Murphy (1953) (Fig. 2 to 5) while others showed irregular hyperplasia of transitional epithelium with some mitotic activity (Fig. 6, 7). A few prostatic ducts and acini near the urethra were also involved (Fig. 9, 10). The epithelium of the prostatic urethra also showed localised patches of irregular epithelium in which the normal arrangement of the cells was lost and the nuclei deeply staining and varying in shape and size. The cytoplasm in some cells was vacuolated (Fig. 11 to 13), forming a halo around a large hyperchromatic nucleus. The sub-epithelial connective tissue was congested and infiltrated with round cells. The picture here is that of Bowen's disease (Bowen, 1912; Stout, 1939) of the urinary tract, as described by Melicow and Hollowell (1952). The remainder of the prostatic tissue removed showed benign nodular hyperplasia. There was no invasion of the stroma by malignant epithelium. Some of the malignant cells in the peri-urethral glands and surface epithelium (but not the vacuolated "Bowen cells") contained P.A.S.-staining material which was not removed by salivary digestion (Fig. 5 and 13).

### DISCUSSION

Melicow and Hollowell (1952), in a report on thirty cases of Bowen's disease of the urinary tract, describe three cases in which lesions were found in the prostatic urethra. One of these was also associated with a large infiltrating carcinoma of bladder. Although occurring in the prostate it seems likely that lesions of this type are probably due to a carcinogenic stimulus affecting transitional epithelium and are probably more closely related to the transitional cell tumours of the urinary tract.

A number of different names, depending on the anatomical site or minor changes in the clinical appearances, have been given to lesions with histologically similar appearances: Paget's disease, Queyrat's erythroplasia, Bowen's disease,

### EXPLANATION OF PLATES

FIG. 1.—A group of large hyperchromatic cells with irregular nuclei in the epithelium of the prostatic urethra. H. & E. × 550.

FIG. 2.—Similar cells in a peri-urethral gland. H. & E. × 550.

FIG. 3.—A group of affected peri-urethral glands. H. & E. × 85.

FIG. 4.—Another group of affected peri-urethral glands; some of the cells lie in clear spaces. H. & E. × 170.

FIG. 5.—A section from the same block as the preceding figure, stained by the periodic acid-Schiff method. Some of the cells contain mucoid material (black), but the clear spaces seen in Fig. 4 do not have this appearance. × 170.

FIG. 6.—Irregular hyperplasia of transitional epithelium in some peri-urethral glands. H. & E. × 85.

FIG. 7.—A high power field from Fig. 6 showing nuclear irregularity. One cell is in mitosis. H. & E. × 1250.

FIG. 8.—An affected prostatic duct is seen at the top. One side is lined by normal columnar epithelium. H. & E. × 85.

FIG 9.—An involved prostatic acinus. H. & E. × 100.

FIG. 10.—A high-power field from the area marked in Fig. 9. The hyperplastic cells are covered by the low columnar epithelium of the acinus. H. & E. × 550.

FIG. 11.—Two localised patches of thickened irregular epithelium in which vacuolated cells can just be made out. H. & E. × 75.

FIG. 12.—A high-power field from the plaque on the right, showing several clear "Bowen cells" in the epithelium. H. & E. × 410.

FIG. 13.—A similar area to Fig. 12. The superficial cells contain a little mucoid material but the Bowen cells do not. P.A.S. stain. × 410.

1

2

3

4

5

Franks and Chesterman.

6

7

8

9

10

Franks and Chesterman.

11

12

13

carcinoma *in situ*, etc. These lesions are commonly found in the skin and less often in the mucous membranes (glans penis, lip, vulva, vagina, tongue, nares, uvula, vocal cord, palate, tonsils (Stout, 1939 ; Cipollaro and Foster, 1940) and urinary tract (Melicow and Hollowell, 1952)). The essential histological features in the skin, as described by many workers, and in the mucosal lesions are similar but the changes are often not so distinctive in the mucous membranes (Stout, 1939).

Paget's disease of the nipple can be separated from this group in that it is almost invariably associated with an adenocarcinoma of the underlying breast. In other sites it would seem preferable to restrict the term " Paget's disease " to epithelial lesions which are also associated with carcinoma of an underlying gland.

The other lesions in the group (Bowen's disease, etc.) may also be associated with infiltrating carcinomatous lesions, but in these cases the carcinomas are almost always squamous (Stout, 1939) or in the urinary tract, transitional celled (Melicow and Hollowell, 1952) and arise from the surface epithelium. One further point of interest is that mucosal Bowen's disease is much more frequently associated with invasive tumours (40 per cent of cases) as compared with the skin lesions (2 to 3 per cent) (Stout, 1939). Fennell and Castleman (1955) suggest that these lesions are cytologically malignant and should be considered as a form or stage of actual cancer (carcinoma *in situ* or pre-invasive cancer) rather than a pre-cancerous lesion.

### SUMMARY

A case of intra-epithelial carcinoma of prostatic urethra, peri-urethral glands and prostatic ducts associated with a carcinoma of the bladder, is reported. If an eponymic title to this type of lesion is required, " Bowen's disease of urinary epithelium " is preferable to " Paget's disease of the prostate ", but a simple descriptive title, e.g. pre-invasive cancer or cancer *in situ* is preferable to either.

Our thanks are due to Dr. J. Bamforth, who originally suggested the diagnosis in this case.

### REFERENCES

Bowen, J. T.—(1912) *J. cutan. Dis.*, **30**, 241.
Cipollaro, A. C. and Foster, P. D.—(1940) *N.Y. St. J. Med.*, **40**, 264.
Fennell, R. H., Jr., and Castleman, B.—(1955) *New Engl. J. Med.*, **252**, 985 and 1032.
Inglis, K.—(1936) ' Paget's Disease of the Nipple ; and its Relation to Surface Cancers and Precancerous States in General.' London (Oxford University Press).
*Idem.*—(1946) *Amer. J. Path.*, **22**, 1.
Melicow, M. M. and Hollowell, J. W.—(1952) *J. Urol.*, **68**, 763.
Muir, R.—(1927) *J. Path. Bact.*, **30**, 451.—(1939) *Ibid.*, **49**, 299.
Ortega, L. G., Whitmore, W. F. Jr., and Murphy, A. I.—(1953) *Cancer*, **6**, 898.
Plachta, A. and Speer, F. D.—(1954) *Ibid.*, **7**, 910.
Stout, A. P.—(1939) *N.Y. St. J. Med.*, **39**, 801.
Weiner, H. A.—(1937) *Amer. J. Cancer*, **31**, 373.
Whorton, C. M. and Patterson, J. B.—(1955) *Cancer*, **8**, 1009.